# Comorbidities in patients with Familial Mediterranean Fever

**DOI:** 10.1186/1546-0096-13-S1-P116

**Published:** 2015-09-28

**Authors:** B Balci-Peynircioğlu, ZS Arıcı, E Avcı, ED Batu, E Arslanoğlu, B Çağlarsu, O Karadağ, U Kalyoncu, Y Bilginer, A Düzova, E Yılmaz, S Özen

**Affiliations:** 1 Hacettepe University, Medical Biology, Ankara, Turkey; 2 Hacettepe University, Pediatric Rheumatology, Ankara, Turkey; 3 Hacettepe University, Rheumatology, Ankara, Turkey

## Introduction

Familial Mediterranean Fever (FMF) is the most common periodic fever syndrome, characterized by recurrent fever, serositis attacks. There are limited data on comorbidities seen in patients with FMF.

## Objective

Our objective was to evaluate comorbidities among individuals with FMF in a large cohort.

## Methods

We used Hacettepe University- Department of Medical Biology's genetic database records of 5636 FMF patients for MEFV mutation. 1998 patients among this group who are followed by rheumatologists in our hospital, with homozygous and compound heterozygous MEFV mutations were included in the study. We analyzed, Hacettepe University clinical records.

## Results

The mean age was 27,5±16 (1-86) years. Our hospital mean follow-up period was 48,5±48 (1-352) months. 1343 patients (67,2%) had no comorbidities. 655 patients (32.8%) had comorbidities. Comorbidities were as follow: Appendectomy 30 (4,6%), cholecystectomy 20 (3,1%), acute rheumatic fever (2,4%), ankylosing spondylitis 155 (23,7%), juvenile idiopathic arthritis 31 (4,6%), rheumatoid arthritis 10 (1,5%), renal amyloidosis 54 (8,2%), intestinal amyloidosis 4 (0,6%), chronic renal failure 33 (5%), Behçet's disease 1 (0,2%), Henoch-Schönlein purpura 25 (3,8%), osteoporosis 77 (11,8%), celiac disease 5 (0,8%), inflammatory bowel disease 16 (2,4%), hepatosplenomegaly 33 (5%), hepatosteatosis 20 (3,1%), hypertension 53 (8,1%), polyarteritis nodosa 9 (1,4%), epilepsy 7 (1,1%), multiple sclerosis 5 (0,8%), PFAPA 4 (0,6%), uveitis 1 (0,2%), asthma 6 (0,9%), IgA deficiency 4 (0,6%), thalassemia trait 8 (1,2 %), thalassemi major 1 (0,2 %), systemic lupus erythematosus 5 (0,8 %), sjogren syndrome 2 (0,4%), autoimmune hepatitis 2 (0,4%), autoimmune hemolytic anemia 2 (0,4%) …

Homozygous M694V frequency in patients with comorbidities was 42,7% while it was 34,4% in those without comorbidities. M694V/M694V mutation in patients with comorbidities was significantly more frequent.

## Conclusion

These comorbidities can be classified in 3 groups: those comorbidities directly related to FMF such as amyloidosis, the second being comorbidities that may be incidental such as IgA deficiency. The last group of comorbidities were those associated with FMF due to increased innate inflammation such as PAN, PFAPA.

It is important to establish the relationship between different diseases’ phenotypes and pathologies underlying cellular functions. Thus the results of our study would lead to improved clinical care and epidemiology in FMF.

